# Assessment of variables associated with prolonged admission duration in children with *Mycoplasma pneumoniae* pneumonia

**DOI:** 10.1111/crj.13549

**Published:** 2022-10-07

**Authors:** Myongsoon Sung, Eui Jeong Roh, Eun Sil Lee, Ji Young Lee, Hyo‐Bin Kim, Youngmin Ahn, Byung Wook Eun, Ja Kyoung Kim, Hyoung Young Kim, Sung‐Su Jung, Minji Kim, Eun Kyeong Kang, Eun‐Ae Yang, Soo Jin Lee, Yang Park, Ju‐Hee Seo, Eun Lee, Eun Seok Yang, Hyung Min Cho, Meeyong Shin, Hai Lee Chung, Yoon Young Jang, Bong Seok Choi, Hyeona Kim, Jin‐A Jung, Seung Taek You, Mi‐Hee Lee, Jin Tack Kim, Bong Seong Kim, Yoon Ha Hwang, Jung Yeon Shim, Hyeon‐Jong Yang, Man Yong Han, Hae Young Yew, Dong Hyeok Kim, Sang Oun Jeong, Kyujam Whang, Eunjoo Lee, You Hoon Jeon, Eun Hee Chung

**Affiliations:** ^1^ Department of Pediatrics Soonchunhyang University Hospital Gumi South Korea; ^2^ Department of Pediatrics Chungnam National University Hospital Daejeon South Korea; ^3^ Department of Pediatrics Hallym University Chuncheon Sacred Heart Hospital Chuncheon South Korea; ^4^ Department of Pediatrics Inje University Sanggye Paik Hospital Seoul South Korea; ^5^ Department of Pediatrics Eulji University Hospital Seoul South Korea; ^6^ Department of Pediatrics Kangwon National University School of Medicine Chuncheon South Korea; ^7^ Department of Pediatrics Pusan National University Children's Hospital, Pusan National University School of Medicine Yangsan South Korea; ^8^ Department of Pediatrics, Chungnam University Sejeong Hospital Chungnam National University College of Medicine Sejong South Korea; ^9^ Department of Pediatrics Dongguk University Ilsan Hospital Goyang South Korea; ^10^ Department of Pediatrics, Daejeon St. Mary's Hospital, College of Medicine The Catholic University of Korea Daejeon South Korea; ^11^ Department of Pediatrics Eulji University Hospital Daejeon South Korea; ^12^ Department of Pediatrics Wonkwang University School of Medicine Iksan South Korea; ^13^ Department of Pediatrics Dankook University College of Medicine Cheonan South Korea; ^14^ Department of Pediatrics Chonnam National University Hostpital, Chonnam National University Medical School Gwangju South Korea; ^15^ Department of Pediatrics College of Medicine, Chosun University, Chosun University Hospital, Gwangju, South Korea Gwangju South Korea; ^16^ Department of Pediatrics Presbyterian Medical Center Jeonju South Korea; ^17^ Department of Pediatrics Soonchunhyang University Bucheon Hospital Bucheon South Korea; ^18^ Department of Pediatrics Daegu Catholic University Medical Center Daegu South Korea; ^19^ Department of Pediatrics School of Medicine, Kyungpook National University Daegu South Korea; ^20^ Department of Pediatrics Donga‐A University College of Medicine Busan South Korea; ^21^ Department of Pediatrics Incheon Medical Center Incheon South Korea; ^22^ Department of Pediatrics College of Medicine, The Catholic University of Korea, Uijeongbu St. Mary's Hospital Uijeongbu South Korea; ^23^ Department of Pediatrics University of Ulsan College of Medicine, Gangneung Asan Hospital Gangneung South Korea; ^24^ Department of Pediatrics Busan ST. Mary's Hospital Busan South Korea; ^25^ Department of Pediatrics Sungkyunkwan University School of Medicine, Kangbuk Samsung Hospital Seoul South Korea; ^26^ Department of Pediatrics Soonchunhyang University Hospital Seoul South Korea; ^27^ Department of Pediatrics, CHA Bundang Medical Center CHA University School of Medicine Seongnam South Korea; ^28^ Department of Pediatrics Kogel Hospital Daejeon South Korea; ^29^ Divison of Bacterial Diseases Bureau of Infectious Disease Diagnosis Control, Korea Disease Control and Prevention Agency (KDCA) Sejong South Korea; ^30^ Department of Pediatrics Inje University Haeundae Paik Hospital Busan South Korea; ^31^ Department of Pediatrics Hallym University Dongtan Sacred Heart Hospital Hwasung South Korea; ^32^ Department of Pediatrics Chungnam National University College of Medicine Daejeon South Korea

**Keywords:** aspartate aminotransferase, children, *M. pneumoniae*, macrolide‐resistant *M. pneumoniae*, neutrophil portion

## Abstract

**Introduction:**

Macrolide‐resistant *Mycoplasma pneumoniae* (MRMP) has become prevalent in children. This study investigated the clinical and laboratory variables of MRMP and macrolide‐sensitive *M. pneumoniae* (MSMP) and identified factors associated with prolonged hospital admission in children.

**Methods:**

A prospective multicenter study was conducted in 1063 children <18 years old in July 2018–June 2020. The 454 had a positive *M. pneumoniae* polymerase chain reaction assay.

**Results:**

Most subjects had MRMP (78.4%), and all mutated strains had the A2063G transition. We defined MRMP* (*n* = 285) as MRMP pneumonia requiring admission and MSMP* (*n* = 72) as MSMP pneumonia requiring admission. Patients with MRMP pneumonia were older, more likely to have segmental/lobar pneumonia, and had more febrile days than those with MSMP pneumonia. C‐reactive protein (CRP), lactate dehydrogenase (LDH), and percentage neutrophils were more strongly associated with MRMP* than MSMP* groups. Percentage neutrophils, CRP, and alanine aminotransferase significantly changed between admission and follow‐up measurements in patients with MRMP* (*P* < 0.05). The duration of admission positively correlated with the number of febrile days after initiation of antibiotic medication and laboratory variables (white blood cell count, CRP, and aspartate aminotransferase [AST]) (*P* < 0.05). Random forest analysis indicated that the number of febrile days after initiation of antibiotic medication, AST, and percentage neutrophils at admission was over five.

**Conclusions:**

This study indicated that children with *M. pneumoniae* pneumonia with a higher number of febrile days after initiation of antibiotic medication, AST, and percentage neutrophils at admission were more likely to have prolonged admission duration.

## Graphical abstract

- Macrolide‐resistant *Mycoplasma pneumoniae* (MRMP) has become prevalent in children.
- Children with *M. pneumoniae* pneumonia with a higher number of febrile days after initiation of antibiotic medication, AST, and percentage neutrophils at admission were more likely to have prolonged admission duration.

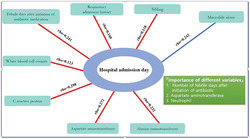

**Abbreviation list**
ALT: alanine aminotransferase
AST: aspartate aminotransferase
CAP: community‐acquired pneumonia
CRP: C‐reactive protein
LDH: lactate dehydrogenase
*M. pneumoniae*
: *Mycoplasma pneumoniae*
MRMP: macrolide‐resistant *M. pneumoniae*
MRMP*: macrolide‐resistant *M. pneumoniae* requiring admission
MSMP: macrolide‐susceptible *M. pneumoniae*
MSMP*: macrolide‐susceptible *M. pneumoniae* requiring admission
OR: odds ratio
PCR: polymerase chain reaction
WBC: white blood cell

## 1 INTRODUCTION

*Mycoplasma pneumoniae* (*M. pneumoniae*) is a significant cause of community‐acquired pneumonia (CAP) in children and adults.^1^, ^2^ *M. pneumoniae* infection is usually benign, has mild symptoms, and often causes mild to moderate pneumonia. However, it can be associated with more severe and life‐threatening diseases and a wide array of extrapulmonary manifestations, which occur in approximately 20%–25% of infected children.^3^ According to an epidemic of *M. pneumoniae* pneumonia reported in 2010–2013, an increasing number of patients with *M. pneumoniae* infection were admitted to the intensive care unit.^4^ Moreover, over 18% of cases in children require hospitalization,^5^ and an estimated two million cases of adult *M. pneumoniae*‐related pneumonia occur annually, resulting in approximately 100 000 hospitalizations in the United States.^6^

Macrolides are used as the first‐line antibiotics in children, but macrolide‐resistant *M. pneumoniae* (MRMP) pneumonia has recently become prevalent in these areas, China, Japan, and South Korea.^7^ Patients with MRMP pneumonia have more severe clinical characteristics, such as extended hospitalization, fever, and antibiotic treatment duration, than patients with macrolide‐sensitive *M. pneumoniae* (MSMP) pneumonia.^8^, ^9^, ^10^

However, there has been controversy regarding differences in clinical characteristics, including hospital admission and laboratory markers, between MRMP and MSMP pneumonia. One study^10^ showed that patients with MRMP pneumonia are at higher risk of fever lasting for >48 h after macrolide treatment (odds ratio [OR], 21.24), and an increased proportion of patients require a second‐line treatment (OR, 4.42). Meanwhile, some other studies have demonstrated that MRMP pneumonia might respond to macrolide treatment, and there was no difference in clinical or laboratory variables between MRMP and MSMP pneumonia.^9^, ^11^, ^12^ Additionally, coinfection with *M. pneumoniae* and other respiratory pathogens are common, but it is not well‐known whether coinfection is related to the severity of illness.^13^

The complicated relationship between hospital admission and these different factors and their relative importance remains unclear in children with *M. pneumoniae* pneumonia. In addition, previous studies of pediatric populations were limited by being performed at a single center, being based in one region, or the incomplete or lack of analysis of prolonged length of hospitalization. Thus, the present study aimed to evaluate children's clinical and laboratory variables with MRMP or MSMP pneumonia and identify factors associated with prolonged hospital stays from 31 centers across six provinces. Also, we investigated the importance of these different factors and laboratory variables, including respiratory virus coinfection, in children with *M. pneumoniae* pneumonia.

## 2 MATERIALS AND METHODS

### 2.1 Participants

A prospective, multicenter study was conducted on children younger than 18 between July 2018 and June 2020. A cooperative hospital monitoring network was established across 31 secondary and tertiary hospitals in Korea. Hospital specialists diagnosed 1063 children with CAP during the study period. The diagnosis of pneumonia was based on both physical examination and radiologic assessments performed in each hospital by respiratory and allergy specialists.

**FIGURE 1:**
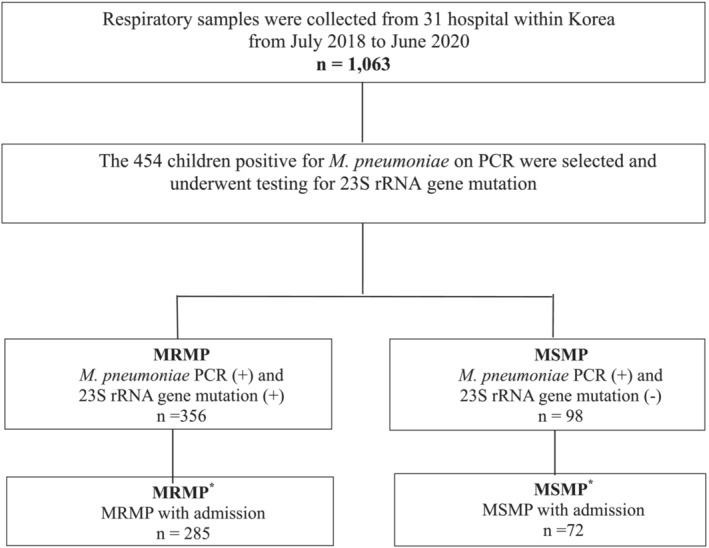
Study population. MRMP, macrolide‐resistant *Mycoplasma pneumoniae* ; MSMP, macrolide‐susceptible *M. pneumoniae* ; PCR, polymerase chain reaction

Among 1063 children, 454 eligible patients had positive *M. pneumonia* results from a polymerase chain reaction (PCR) assay. The children with a history of antibiotic use within 5 days were excluded from the present study. In the present study, we defined MRMP as a positive PCR result with mutations at residues 2063 and 2064 (*n* = 356) and MSMP as a positive PCR result with no mutations at residues 2063 and 2064 (*n* = 98) (Figure 1). We defined MRMP* (*n* = 285) as MRMP infection requiring admission and MSMP* (*n* = 72) as MSMP infection requiring admission (Figure 1).

### 2.2 Demographic, clinical, and laboratory data

Medical records were reviewed to collect general information about the children (e.g., sex, birth date, height, weight, admission history, family history of allergic disease, the prescribed antibiotic medication type at admission first day or outpatient clinic, demographic characteristics, and chest radiograph findings). Also, we evaluated the laboratory variables of children, such as the total white blood cell (WBC) count, percentage of neutrophils, C‐reactive protein (CRP), and virus co‐infection. All blood samples were obtained at the time of admission.

### 2.3 Nucleic acid extraction and PCR analysis

Samples were obtained from the sputum, bronchoalveolar lavage, nasopharyngeal aspiration, or nasopharyngeal swabs within 24 h after enrollment of hospital admission or outpatient clinic visit. All specimens containing *M. pneumoniae* DNA were stored at −70°C before testing. According to the manufacturer's instructions, *M. pneumoniae* was detected using PCR within 48 h and confirmed using the AllplexTMPneumoBacter Assay (Seegene, Seoul, Korea). Nucleic acid was extracted from 1 ml of the sample and purified. The cyclic temperature settings were 95°C for 2 min, 60°C for 1 min, and 72°C for 2 min and amplified for 35 cycles at 72°C for 7 min.

Samples that were positive following PCR amplification were screened for point mutations in domain V of 23S rRNA associated with macrolide resistance. The primers targeted residues 1949–1968 (forward) and 2148–2167 (reverse) in domain V of 23S rRNA (Myco23S‐V‐F AGT CGG GTA AAT TCC GTC CC and Myco23S‐V‐R CGC ATC AAC AAG TCC TAG CG). The PCR products were analyzed using the BigDye® Terminator v3.1 Cycle Sequencing Kit and an ABI 3500 XL series (Applied Biosystems, Thermo Fisher Scientific, Foster City, CA, USA).

### 2.4 A viral pathogen

For multiplex RT‐PCR, viral genomic RNA and DNA were extracted from a total volume of 1 μl of the sample using the guanidinium thiocyanate extraction method. The lysis buffer included 500 molecules of the cloned amplified product used as an internal control in each reaction tube and excluded false‐negative results. Three independent multiplex reverse transcription nested RT‐PCR assays able to detect 1–10 copies of viral genomes were performed. A nested RT‐PCR was performed using a specific primer for PIV (1, 2, 3, 4), ADV, HEV, and HMPV; another nested RT‐PCR was prepared with specific primers for HRV (A, B, C), CoV (229E, NL63, OC43) and BoV (1, 2, 3, 4) and a third nested RT‐PCR was performed using specific primers for RSV (A, B) and IFV (A, B, subtype H1, H3, H1pdm09) using the AllplexTM Respiratory Panel (Seegene, Seoul, South Korea).

### 2.5 Possible causes of prolonged length of hospitalization in children with *M. pneumoniae* pneumonia

We analyzed specific items that had significant associations with *M. pneumoniae* pneumonia in previous studies (e.g., age, sex, febrile days after initiation of antibiotic medication, MRMP, and laboratory variables including viral coinfection)^1^, ^2^, ^3^, ^4^, ^5^, ^6^, ^7^, ^8^, ^9^, ^10^, ^11^, ^12^, ^13^ were also considered. These items also included general characteristics of each child (admission season; respiratory admission history; siblings; daily center/kindergarten use; body mass index; and treatment with macrolides alone, tetracycline, and/or quinolone).

### 2.6 Ethics statement

The study protocol was approved by the Institutional Review Board and Ethics Committee of Chungnam National University Hospital (IRB No 2019‐07‐037) and all participating medical centers. Written informed consent was obtained from the parents or guardians of all participants following a detailed explanation of the study.

### 2.7 Statistical analysis

Statistical analysis was performed using IBM SPSS Statistics (version 23.0; IBM Corp., Armonk, NY, USA) and R version 2.8.134 (Vienna, Austria). The experimental results are presented as the mean ± standard deviation, whereas categorical data are reported as numbers (percentages). Inter‐group comparisons were performed using the Mann–Whitney test or Kruskal–Wallis test for continuous variables and the chi‐squared test or Fisher's exact test for categorical variables.

Random forest analyses were performed using R, and a variable importance ranking of each random forest was created. The mean decreases accuracy (MDA) and Gini were calculated to model accuracy by permuting each feature's value and measuring each variable's importance in estimating the target variable. To ease interpretation of the graphical displays, a suitable ordering of the components was identified using seriation.^14^ The random forest can analyze data with a non‐linear trend or extrapolation without importance. Moreover, this analysis easily and intuitively demonstrates the quantitative priorities of each risk factor.^15^ Also, we used false discovery rate (FDR) for adjusted *P*‐value to adjust for multiplicity. Statistical significance was defined as a *P*‐value below 0.05.

## 3 RESULTS

### 3.1 Baseline characteristics of the study participants

The clinical course of the 454 patients with positive *M. pneumoniae* PCR results is summarized in Table 1. All participating children (*n* = 454 [100%], male/female = 221/233, mean age = 6.89 ± 3.77 years) were divided into two groups: MRMP (*n* = 356, 78.4%) and MSSP (*n* = 98, 21.6%). All isolates in the MRMP group (*n* = 356) had A2063G point mutations.

**TABLE 1:** Baseline characteristics of children with *Mycoplasma pneumoniae* pneumonia

Characteristics	MRMP *n* = 356 (100)	MSMP *n* = 98 (100)	*P*	MRMP^*^ *n* = 285 (100)	MSMP^*^ *n* = 72 (100)	*P*	*P* ^a^	*P* ^b^
**Sex (male/female)**	163/193	58/40	**0.019**	144/141	23/49	**0.005**	0.234	**<0.001**
**Mean age (years)**	7.34 ± 3.47	5.28 ± 4.35	**<0.001**	7.47 ± 3.30	5.17 ± 4.35	**<0.001**	0.624	0.887
**Age (years)**			**<0.001**			**<0.001**	0.722	0.888
≤2	28 (7.9)	34 (34.7)		16 (5.6)	27 (37.5)			
3–5	83 (23.3)	22 (22.4)		67 (23.5)	13 (18.1)			
6–11	204 (57.3)	33 (33.7)		170 (59.6)	24 (33.3)			
≥12	41 (11.5)	9 (9.2)		32 (11.2)	8 (11.1)			
**Total febrile day before admission (day)**	5.43 ± 3.02	3.54 ± 2.90	**<0.001**	5.83 ± 2.83	3.78 ± 2.76	**<0.001**	0.098	0.495
**Daily center/kindergarten, *n* (%)**				283 (99.3)	57 (79.2)	**<0.001**		
**Sibling, *n* (%)**				274 (96.1)	49 (68.1)	<0.001		
**Segmental or lobar pneumonia**	256 (72.0)	45 (45.9)	**<0.001**	251 (88.1)	42 (58.3)	**<0.001**	**<0.001**	0.110
**Antibiotics medication**								
**Initial antibiotics, *n* (%)**	335 (94.1)	91 (92.9)	0.650	284 (99.6)	70 (97.2)	**0.007**	**<0.001**	0.183
**Prescribed antibiotics, *n* (%)**								
**Macrolide**								
Macrolide alone	102 (28.7)	27 (27.6)	0.831	85 (29.8)	20 (27.8)	0.427	**<0.001**	0.554
Macrolide + cephalosporin	61 (17.1)	24 (24.5)	0.098	58 (20.4)	2 (2.8)	**<0.001**	0.298	**<0.001**
Macrolide + β‐lactams	55 (15.4)	7 (7.1)	**0.034**	28 (9.8)	24 (33.3)	**<0.001**	**0.003**	**<0.001**
**Tetracycline or quinolone**	72 (20.2)	11 (11.2)	**0.041**	65 (22.8)	11 (15.3)	0.107	0.439	0.492

*Note*: Values are presented as numbers (%) and mean ± standard deviation. Numbers in bold indicate significant differences (*P* < 0.05). Scale variables were analyzed using the chi‐squared test or Fisher exact test, and continuous variables were analyzed using Student's *t*‐test or Mann–Whitney *U*‐test.

Abbreviations: MRMP, macrolide‐resistant *M. pneumoniae*; MRMP*, macrolide‐resistant *M. pneumoniae* with admission; MSMP, macrolide‐susceptible *M. pneumoniae*; MSMP*, macrolide‐susceptible *M. pneumoniae* with admission; NA, not evaluated.

^a^ Compared with MRMP and MRMP*.

^b^ Compared with MSMP and MSMP*.

Compared with the MSMP group, the mean age of children, proportion of female participants, and total number of febrile days was higher in the MRMP group (*P* < 0.05). The proportion of patients who had segmental or lobar pneumonia or received tetracycline or quinolone treatment was higher in the MRMP group (*P* < 0.05) (Table 1).

### 3.2 Comparison of the clinical characteristics between MSMP* and MRMP* groups

The clinical course and laboratory findings of 357 patients with positive *M. pneumoniae* PCR results upon hospital admission are summarized in Tables 1 and 2. According to the 23S rRNA gene mutation results, the 357 patients were divided into MRMP* (*n* = 285) and MSMP* (*n* = 72) groups (Figure 1).

**TABLE 2:** Clinical characteristics of children in the MRMP* and MSMP* groups (*n* = 357)

Characteristics	MRMP* *n* = 285 (100)	MSMP* *n* = 72 (100)	Total *n* = 357 (100)	*P* ^a^
**BMI**	18.14 ± 4.24	17.35 ± 3.04	17.98 ± 4.04	0.374
**Respiratory admission history, *n* (%)**	44 (15.4)	20 (27.8)	64 (17.9)	**0.035**
**Hospital admission duration (day)**	7.23 ± 4.30	6.36 ± 2.67	7.06 ± 4.04	0.374
**Incident season**				0.376
Spring (Mar, Apr, May)	29 (10.2)	5 (6.9)	34 (9.5)	
Summer (Jun, Jul, Aug)	3 (1.1)	2 (2.8)	5 (1.4)	
Autumn (Sep, Oct, Nov)	127 (44.6)	38 (52.8)	165 (46.2)	
Winter (Dec, Jan, Feb)	126 (44.2)	27 (37.5)	153 (42.9)	
**Febrile days after initiation of antibiotic medication (day)**	2.44 ± 2.23	1.58 ± 1.84	2.27 ± 2.18	**0.007**
**Hypoxia (SpO**_**2**_ **< 93%)**	15 (5.3)	2 (2.8)	17 (4.8)	0.376
**Pleural effusion, *n* (%)**	36 (12.6)	2 (2.8)	38 (10.6)	**0.035**

*Note*: Values are presented as numbers (%) and mean ± standard deviation. Numbers in bold indicate significant differences (*P* < 0.05). Scale variables were analyzed using the chi‐squared test or Fisher exact test, and continuous variables were analyzed using Student's *t*‐test or Mann–Whitney *U*‐test.

Abbreviations: MRMP, macrolide‐resistant *Mycoplasma pneumoniae*; MRMP*, macrolide‐resistant *M. pneumoniae* with admission; MSMP, macrolide‐susceptible *M. pneumoniae*; MSMP*, macrolide‐susceptible *M. pneumoniae* with admission.

^a^ *P* was analyzed using a false discovery rate for multiplicity.

Compared with the MRMP group, the proportion of participants with segmental or lobar pneumonia and the initial antibiotics and macrolide treatment was higher in the MRMP* group. The proportion of patients treated with a macrolide plus cephalosporin or β‐lactams was different between the MSMP and MSMP* groups (*P* < 0.05) (Table 1).

Meanwhile, the mean age of children was higher, and the total number of febrile days and number of febrile days after initiation of antibiotic medication were higher in the MRMP* group, compared with the MSMP* group. The proportion of patients who attended a daily center/kindergarten had a sibling was also higher in the MRMP* group (*P* < 0.05) (Tables 1 and 2). In addition, segmental or lobar pneumonia, respiratory admission history, and the incidence of pleural effusion were significantly different between the MRMP* and MSMP* groups (*P* < 0.05). However, there was no difference in hospital admission days between the two groups (Table 2).

Regarding antibiotics medication, the proportion of patients who received initial antibiotics or macrolide plus cephalosporin or β‐lactam treatment was also higher in the MRMP* group (*P* < 0.05). However, there was no difference in those treated with a macrolide alone, tetracycline, or a quinolone (Table 1).

### 3.3 Comparison of laboratory variables between MSMP* and MRMP* groups

The median CRP and lactate dehydrogenase (LDH) levels in 357 children with *M. pneumoniae* at admission were 5.97 ± 14.28 mg/dl and 303.11 ± 304.07 U/l, respectively, and were significantly different between the MRMP* and MSMP* groups (*P* < 0.001 and *P* = 0.001). The relative proportion of neutrophils was higher in the MRMP* pneumonia group (*P* < 0.001). The relative proportion of respiratory virus coinfection and adenovirus infection was higher in the MSMP* pneumonia group (*P* < 0.05). However, the WBC count at admission did not differ between the two groups (Table 3).

**TABLE 3:** Laboratory findings and comparison at the time of hospital admission and follow‐up in MRMP* and MSMP*

	MRMP*	MRMP*	*P* ^a^	MSMP*	MSMP*	*P* ^b^	*P* ^c^
	Admission (*n* = 285)	Follow‐up (*n* = 145)		Admission (*n* = 72)	Follow‐up (*n* = 27)		
**White blood cell counts (/mm** ^**3**^ **)**	8175.54 ± 3726.25	9646.66 ± 8277.81	0.399	8783.19 ± 3322.15	5570.89 ± 4818.60	**0.022**	0.071
**Neutrophil proportion (%)**	66.77 ± 30.41	61.01 ± 17.00	**0.002**	55.49 ± 16.99	46.49 ± 18.86	0.064	**<0.001**
**C‐reactive protein (mg/dl)**	4.39 ± 5.51	2.86 ± 7.35	**<0.001**	3.53 ± 11.20	4.98 ± 7.47	0.442	**<0.001**
**Lactate dehydrogenase (U/L)**	278.72 ± 299.30	456.81 ± 345.90	**<0.001**	399.64 ± 305.67	301.00 ± 393.73	0.263	**0.001**
**Aspartate aminotransferase (IU/ml)**	41.95 ± 96.69	31.06 ± 17.30	0.764	31.60 ± 11.05	29.60 ± 31. 38	0.061	0.675
**Alanine aminotransferase (IU/ml)**	29.37 ± 77.50	39.14 ± 49.74	**<0.001**	16.42 ± 8.36	29.7 ± 43.91	0.875	0.055
**Respiratory virus coinfection, *n* (%)**	115 (40.4)			43 (59.7)			**0.004**
Rhinovirus	63 (22.1)			11 (15.3)			0.202
Adenovirus	24 (8.4)			16 (22.2)			**0.003**
Respiratory syncytial virus	23 (8.1)			10 (13.9)			0.169

*Note*: Values are presented as numbers (%) or mean ± standard deviation. Numbers in bold indicate significant differences (*P* < 0.05).

Abbreviations: MRMP*, macrolide‐resistant *Mycoplasma pneumoniae* with admission; MSMP*, macrolide‐susceptible *M. pneumoniae* with admission.

^a^ Sampling at admission compared with follow‐up sampling in MRMP* from Wilcoxon signed rank test.

^b^ Sampling at admission compared with follow‐up sampling in MSMP* from Wilcoxon signed rank test.

^c^ Compared with MRMP* and MSMP* were analyzed using Student's t‐test or Mann–Whitney *U*‐test.

Of a total of 357 patients with MRMP* and MSMP*, only 172 children (MRMP*, *n* = 145; MSMP*, *n* = 27) were followed‐up on the day of hospitalization with ≥48 h of fever. On initial and follow‐up tests, laboratory variables, including percentage neutrophils, CRP, LDH, and alanine aminotransferase (ALT), were significantly changed in the MRMP* group (*P* < 0.05). However, no significant differences were observed between admission and before discharge in any of the laboratory variables in MSMP*, except WBC count (*P* < 0.05) (Table 3).

### 3.4 Association of the length of hospitalization with various factors in *M. pneumoniae*

**FIGURE 2:**
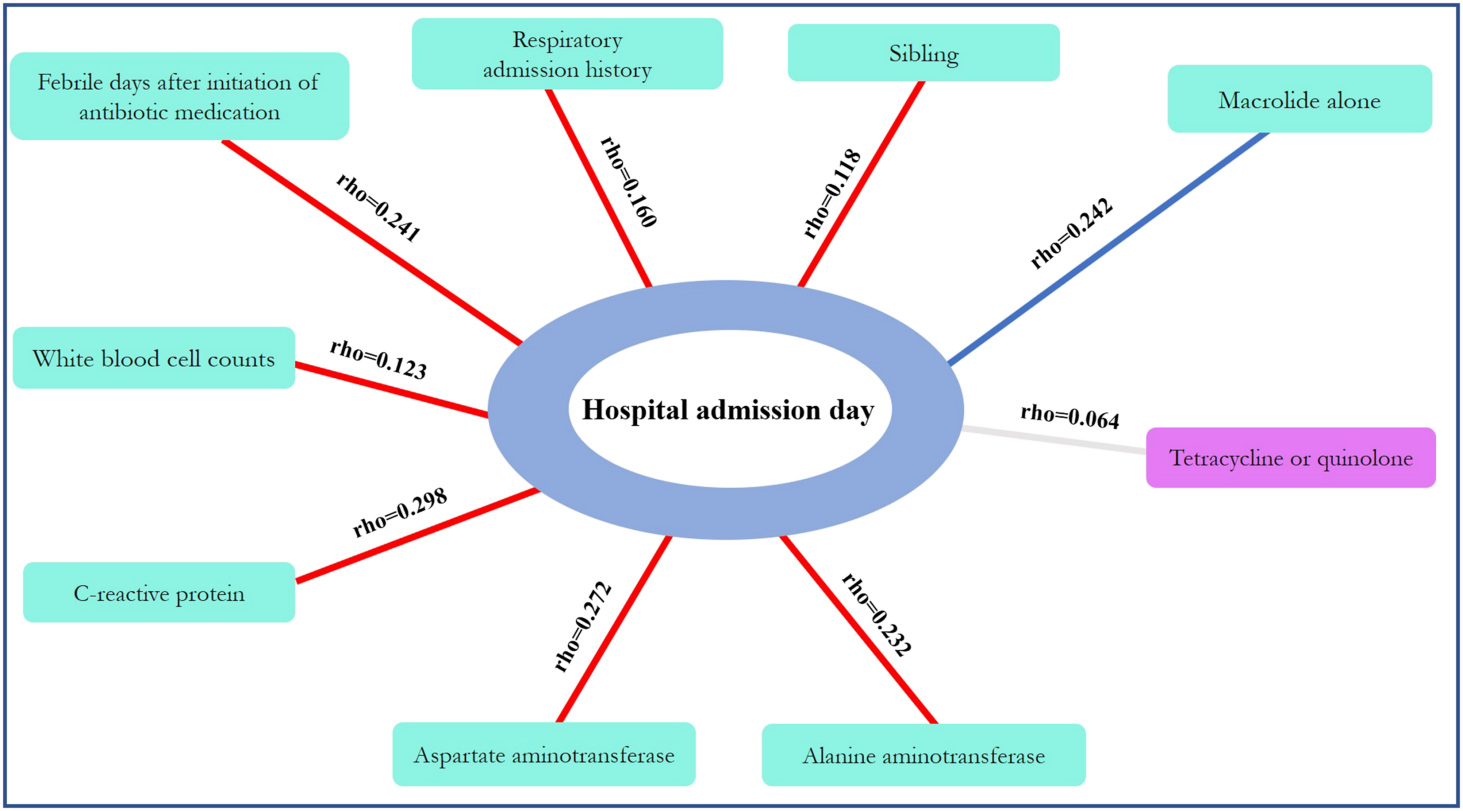
Factors significantly associated with prolonged length of hospitalization (*P* < 0.05, except tetracycline or quinolone). Red line: positive correlation; blue line: negative correlation; Rho: correlation coefficient

Our analysis indicated that a more prolonged length of hospitalization was associated with multiple factors (Figure 2). In particular, length of hospitalization was positively associated with more febrile days after initiation of antibiotic medication, previous history of admission for respiratory disease, having a sibling, and laboratory variables (WBC, CRP, aspartate aminotransferase [AST], and ALT levels) (*P* < 0.05). However, the length of hospitalization was negatively associated with macrolide treatment alone (*P* < 0.05) but not with tetracycline or quinolone treatment (*P* > 0.05).

### 3.5 Importance of different variables in causing the prolonged length of hospitalization in children with *M. pneumoniae* pneumonia

**FIGURE 3:**
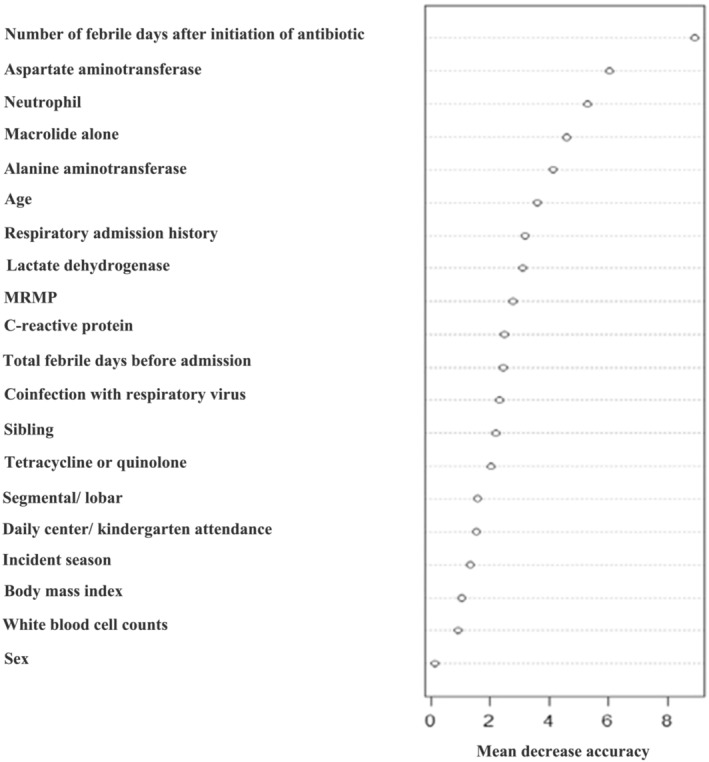
Random forest variable importance plot. Mean decrease accuracy measures the model's performance without each variable. A higher value indicates the importance of different variables on prolonged length of hospitalization in children with *Mycoplasma pneumoniae* . Removal of that variables causes the model to lose accuracy in prediction. MRMP, macrolide‐resistant *M. pneumoniae*

The random forest has the benefit of analyzing data with a non‐linear trend or extrapolation without importance and quickly and intuitively demonstrates the quantitative priorities of each risk factor.^15^ Also, the MDA was calculated to model accuracy by permuting each feature's value and measuring each variable's importance in estimating the target variable in the present study. Thus, we considered all variables simultaneously and used the random forest method to identify the most significant factors causing prolonged hospitalization in children with *M. pneumoniae* (Figure 3).

Figure 3 shows the mean decrease in accuracy for each risk factor, such as more febrile days after initiation of antibiotic medication (MDA, ¼ 8.90), AST levels (6.02), and percentage neutrophils (5.29). These are influential factors related to longer admission days; treatment with a macrolide alone (4.61), previous history of admission for respiratory disease (3.18), and MRMP (2.79) were also influential factors in causing longer hospital admission days in children with *M. pneumoniae* pneumonia.

## 4 DISCUSSION

This multicenter and prospective study identified that 78.4% of the children had the A2063G mutation in domain V of 23S rRNA during Korea's 2018–2020 *M. pneumoniae* pneumonia epidemic. Previous studies have reported that the MRMP rate in 2011 was 51.6–62.9%,^7^, ^16^ but 60%–87% of children were reported to have the A2063G mutation during 2018–2020 in Korea,^10^, ^12^, ^15^, ^16^, ^17^, ^18^ which is in agreement with the findings of the present study. Additionally, we evaluated the differences in clinical and laboratory variables between MRMP and MSMP and various factors related to admission duration in children with *M. pneumoniae* pneumonia, and then ranked the importance of these different factors. Recent studies have demonstrated that patients with MRMP pneumonia may be associated with prolonged fever relative to patients with MSMP pneumonia.^19^, ^20^, ^21^ However, other studies have suggested no significant differences in clinical or radiographic findings.^18^, ^22^, ^23^, ^24^

We found that the MRMP pneumonia was older, had more febrile days, and were more likely to have segmental/lobar pneumonia relative to the MSMP pneumonia, regardless of the admission status. Additionally, the present study showed significant differences in some clinical (previous respiratory admission and febrile days after initiation of antibiotic medication) and radiological characteristics upon admission between the two groups of children with *M. pneumoniae*. A previous Korean study^2^ identified differences in clinical characteristics, such as the number of febrile days after initiation of macrolide treatment and the number of patients with fever lasting >72 h after initiation of macrolides, between the MRMP and MSMP groups, consistent with the findings in this study. In addition, the mean age and age disturbance of children with *M. pneumoniae* pneumonia was higher in the 2015 epidemic than in the 2011 epidemic. However, there was no difference in age between the MRMP and MSMP groups^2^ and another Korean study from 2019–2020.^12^ These differences came from that these studies were conducted in a single center, and fewer participants were included than in our study, which was conducted across 31 centers and included 456 subjects across Korea.

MRMP and MSMP pneumonia have clinical and radiological differences, such as fever duration and lobar pneumonia incidence,^8^, ^9^, ^10^ as shown in the present study. A previous study demonstrated that 72.4% of the patients in the MRMP group had large lesions with radiological manifestations, and a higher incidence of pleural effusion, lobar atelectasis, and consolidation was found in the MRMP group than in the MSMP group.^25^ Because of the direct microbe effect and host immune response, imaging findings can show great diversity, and this significant radiological evidence of lung damage complied with the complicated course of the disease.^25^ Thus, our results indicate that segmental/lobar (88.1%) and pleural effusion (12.6%) were more common in the MRMP group upon admission. Unfortunately, the present study had some regional differences in the number of subjects across provinces. Thus, further objective studies are necessary to ascertain whether older individuals are more susceptible to MRMP pneumonia.

Recent studies have attempted to identify laboratory variables for predicting MRMP. According to previous studies,^25^, ^26^, ^27^ AST, ALT, LDH, and CRP levels and various cytokines are considered potentially valuable biomarkers for severe or refractory pneumonia. Initial immunological insults may induce further inflammation and possibly secondary bacterial invasion, suggesting elevated LDH and CRP levels, and some biomarkers may reflect lung tissue damage and an increased risk of other organ involvement. We found that CRP and LDH levels and percentage neutrophils were more associated with MRMP pneumonia than MSMP pneumonia, agreeing with a previous study^25^; however, LDH levels were higher in the MSMP group. Furthermore, CRP, LDH, ALT, and percentage neutrophils significantly changed in the MRMP group; however, the WBC count significantly changed in the MSMP group. In previous studies, initial and follow‐up tests for laboratory markers including WBC, CRP, LDH, AST, and ALT levels showed statistically significant differences^12^; however, CRP levels did not change after infection with *M. pneumoniae* in children.^28^ However, the follow‐up sample size was small in the previous and present studies, so there is a lack of objectivity due to selection bias.

Previous studies have demonstrated that MRMP pneumonia may have a prolonged length of hospitalization relative to MSMP pneumoniae^19^, ^20^, ^21^; however, other studies have suggested no significant differences in the duration.^22^, ^23^, ^24^ In the present study, there was no significant difference in the length of hospitalization between the MSMP* and MRMP* groups. Coinfection of *M. pneumoniae* and other respiratory pathogens is common.^13^ We found coinfection with other respiratory pathogens in over 40% in both groups, and that coinfection was more strongly associated with MSMP than with MRMP. We postulated that because of the high rate of coinfection, children with MSMP were admitted, and there would be no difference in the length of hospitalization.

One interesting result from this study is that a higher number of febrile days after initiation of antibiotic medication, AST level, and percentage neutrophils were the most critical factors contributing to a prolonged length of hospitalization, of which a number of febrile days after initiation of antibiotic medication was the most potent factor. Furthermore, a macrolide treatment alone, previous history of admission for respiratory disease, and MRMP were influential factors causing the longer length of hospitalization in children with *M. pneumoniae* pnemonia. To the best of our knowledge, this study is the first study of children infected with *M. pneumoniae* to rank the relative importance of different factors on a prolonged length of hospitalization.

This study has a few limitations. First, the investigation period was from July 2018 to June 2020; the number of samples decreased dramatically from February 2020, the beginning of the COVID19 epidemic. Second, there were some regional differences in the number of participants among the six provinces within Korea had. Nevertheless, the strengths of the present study are that we analyzed data from 454 children following a medical review, including radiological findings and blood sampling data, from within Korea over 24 months. Unlike previous studies on this topic, we were able to rank the relative importance of different factors on a prolonged length of hospitalization in children with *M. pneumoniae*.

Our study indicated that children with *M. pneumoniae* pneumonia with a higher number of febrile days after initiation of antibiotic medication, AST levels, and percentage neutrophils were more likely to have prolonged length of hospitalization. Certain factors such as macrolide treatment alone and the MRMP group also affected the probability of extended length of hospitalization. Strategies to predict the length of hospitalization in children with *M. pneumoniae* pneumonia should focus on the factors identified here.

## CONFLICT OF INTEREST

We declare that there are no real or perceived conflicts of interest to declare related with this submission and that we have no links with industry.

## ETHICS STATEMENT

This study was approved by the Institutional Review Board and Ethics Committee of Chungnam National University Hospital (IRB No 2019‐07‐037). The parents of all study participants gave written informed consent before study enrollment. All methods were performed in accordance with the relevant guidelines and regulations.

## AUTHOR CONTRIBUTIONS

MS was involved with data curation. MS, EHJ, and JYS were involved with writing the original draft. And writing—review and editing. All authors contributed equally to this manuscript and were responsible for data analysis and interpretation. All authors drafted the manuscript, provided critical revisions, and approved the final version of the manuscript for submission.

## Data Availability

The data were collected through the surveillance system of Korean Childhood Community Acquired Pneumonia Study Group of Korean Academy of Pediatric Allergy and Respiratory Disease. Data are available from the corresponding authors on reasonable request and with permission of Korean Academy of Pediatric Allergy and Respiratory Disease.
